# The Effect of Tactile Training on Sustained Attention in Young Adults

**DOI:** 10.3390/brainsci10100695

**Published:** 2020-09-30

**Authors:** Yu Luo, Jicong Zhang

**Affiliations:** 1 School of Biological Science and Medical Engineering, Beihang University, Beijing 100083, China; yuluo@buaa.edu.cn; 2 Beijing Advanced Innovation Center for Big Data-Based Precision Medicine, Beihang University, Beijing 100083, China; 3 Beijing Advanced Innovation Centre for Biomedical Engineering, Beihang University, Beijing 100083, China; 4 Beihang University Hefei Innovation Research Institute, Hefei 230013, China

**Keywords:** sustained attention to response task (SART), tactile training, EEG source imaging, sustained attention improvements

## Abstract

Sustained attention is crucial for higher-order cognition and real-world activities. The idea that tactile training improves sustained attention is appealing and has clinical significance. The aim of this study was to explore whether tactile training could improve visual sustained attention. Using 128-channel electroencephalography (EEG), we found that participants with tactile training outperformed non-trainees in the accuracy and calculation efficiency measured by the Math task. Furthermore, trainees demonstrated significantly decreased omission error measured by the sustained attention to response task (SART). We also found that the improvements in behavioral performance were associated with parietal P300 amplitude enhancements. EEG source imaging analyses revealed stronger brain activation among the trainees in the prefrontal and sensorimotor regions at P300. These results suggest that the tactile training can improve sustained attention in young adults, and the improved sustained attention following training may be due to more effective attentional resources allocation. Our findings also indicate the use of a noninvasive tactile training paradigm to improve cognitive functions (e.g., sustained attention) in young adults, potentially leading to new training and rehabilitative protocols.

## 1. Introduction

The lives of young adults nowadays are filled with media and technology multitasking [1]. Previous studies have shown that excessive media multitasking is associated with challenges to the attentional functioning of young adults, such as decreased attention control [1], increased distractibility [2], more mind wandering [3], diminished attention span [4], low personal satisfaction, and poor academic performance [4,5]. Considering the fact that sustained attention is pivotal for efficiently utilizing information, enhancing cognitive functions (e.g., working memory [6], emotion regulation [7]), and ultimately contributing to evolution for survival [8], there is an increasing need for the development of new techniques to improve sustained attention abilities.

Despite the fact that young adults demonstrate superior attention functions compared with old people and clinical populations who are usually more responsive to the benefits of attention training, young adults nevertheless are faced with many real-world attention challenges [9,10]. Exposed to various media multitasking and facing with numerous attention challenges, young adults tend to use prescription stimulants for the improvement of sustained attention and academic performance [11]. Using quantitative and qualitative methodologies, a survey of 1811 undergraduates reported that as many as 43% of college students abused stimulants [12]. The illegal use of stimulants in this population leads to negative outcomes. A meta-analysis and systematic review showed that misuse of stimulant medication was associated with complications (e.g., depression symptoms) and even life-threatening consequences [13]. Additionally, misuse of prescription stimulants was not found to truly enhance sustained attention [11]. Therefore, there is a growing demand for accessible and affordable new ways to improve the attention skills of healthy young people.

Although computer-based exercises and meditation have been developed for attention training, the methods nevertheless may induce mental exhaustion and fatigue [14], or require the access to trained expert facilitators and months of face-to-face meetings [15]. These training methods also do not offer performance feedback during tasks and quantifiable measurements of success [4], which are considered to be important for maintaining participation and long-term compliance [16]. Recent studies have started to develop attention training paradigms based on the sensorimotor modalities. Numerous studies have shown plasticity in the sensory and motor systems [17,18]. However, it remains unclear whether a tactile training can improve visual sustained attention. There are also few electrophysiological studies to investigate the underlying brain activity of the tactile training effects on sustained attention in young adults.

The main purpose of this study was to investigate whether a closed-loop tactile training can improve sustained attention. This has important clinical and practical applications because enhancement of sustained attention via training may not only help young adults, but can also affect children [19,20], those with attention-deficit disorders [9], and special professionals, such as drivers, pilots, and air traffic controllers [21,22]. We explored the tactile training effects on sustained attention as measured by the Math task and SART. In addition to examining the cognitive performance, participants also underwent EEG recordings during the SART, enabling us to identify the event-related potential (ERP) components and disentangle the possible brain sources of such tactile training effects.

## 2. Materials and Methods

### 2.1. Ethics Statement

This study was approved by the Science and Ethical Committee of the School of Biological Science and Medical Engineering of Beihang University, Beijing, China (BM20200170). It was performed following the World Medical Association code of ethics (Declaration of Helsinki) for experiments involving human subjects. Written informed consent was obtained from all participants before the experiment.

### 2.2. Participants

A total of 34 young adults (22.7 ± 1.4 years, sixteen females) participated in the current study. The participants were randomly divided into the experiment (22.5 ± 1.6 years, eight females) and control (22.9 ± 1.1 years, eight females) groups. Independent samples *t*-tests revealed no significant differences regarding age, education or attention level, as measured by the daydreaming frequency subscale [23], between the two groups (Table 1). All participants were recruited through campus forums and from among students of Beihang University. All participants were healthy right-handed native Chinese speakers with normal or corrected-to-normal vision. No participant had a history of somatosensory, neurological, or psychiatric disorder.

### 2.3. Experimental Design

**Figure 1:**
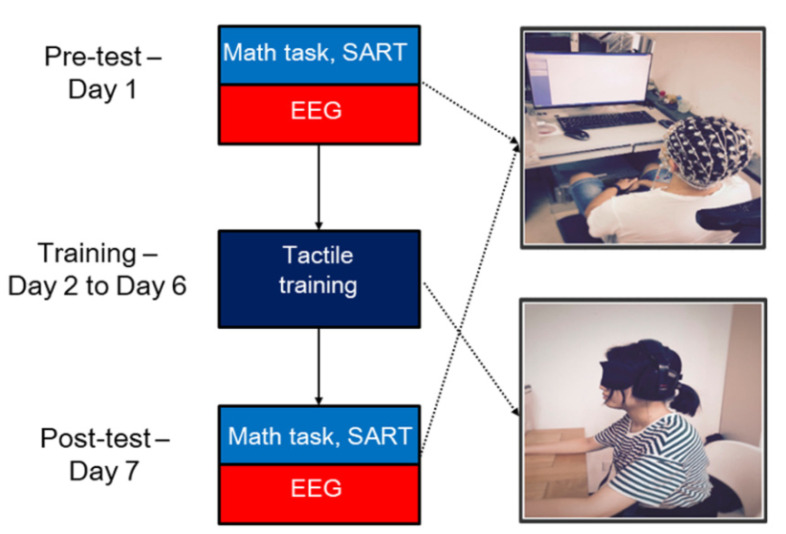
Study paradigm. The entire study paradigm lasted for 7 days including (1) pre-test session: The Math task and the sustained attention to response task (SART) assessments with electroencephalography (EEG) recordings, (2) the tactile training session, and (3) post-test session: The Math task and SART assessments with EEG recordings. From Day 2 to Day 6, participants in the experimental group wore earplugs and an eye mask during the tactile training. On Day 1 and Day 7, participants in the experimental group performed the Math task and SART assessments with EEG recordings. Participants in the control group only underwent the Math task assessment and EEG recordings on Day 1 and Day 7.

Participants in the experimental group underwent the training and test sessions, whereas participants in the control group only underwent the test session. The whole experiment lasted 7 days and was divided into three stages: a pre-test session, a training session, and a post-test session (Figure 1). On the first day, all participants received the SART and Math tasks examination, with EEG recorded simultaneously. The tactile training involved the manipulation of the adaptive fingertip tactile device for approximately 40 min per day over 5 successive days. Participants in the control group did not receive the tactile training and the SART. All participants returned for a follow-up assessment on the final day that was the same as on day 1. Three participants in the experimental group lacked the SART due to their schedules.

### 2.4. The SART

**Figure 2:**
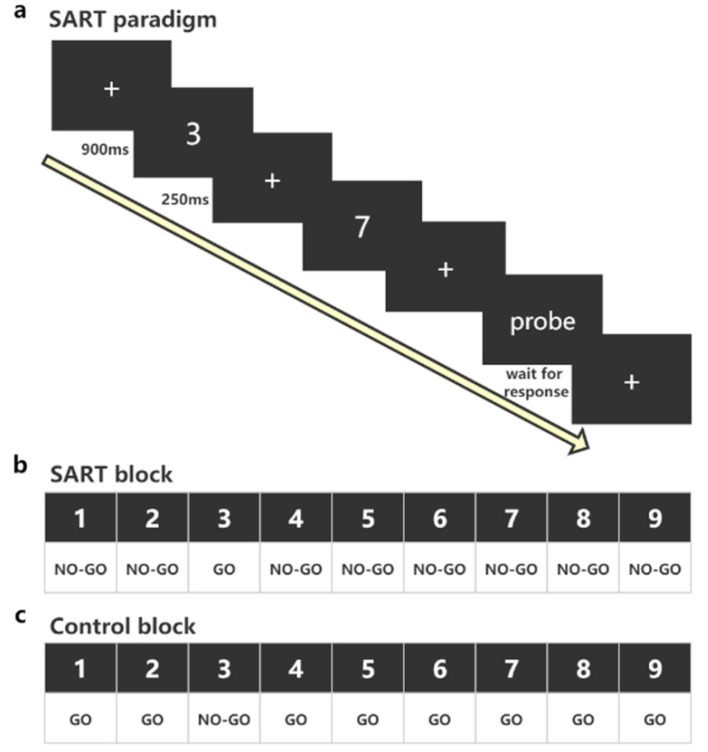
The sustained attention to response task (SART). (**a**) The SART paradigm. For the real-time probe, one of the three questions was presented randomly and waited for the participant to response; (**b**) the target (digit 3) and nontargets (1, 2, 4–9) in the SART block. Participants were required to respond by key press to digit 3 while ignoring all other digits. (**c**) The target (1, 2, 4–9) and nontargets (digit 3) in the Control block. Participants were required to respond by key press to all digits (1, 2, 4–9) except digit 3.

The SART (Figure 2) is a computerized go/no-go task widely used to predict the frequency of daily attention lapses and investigate sustained attention in health and disease [24]. In the current study, the SART was used as the test task in the pre-test and post-test sessions. The SART consists of a SART block and a Control block. Both blocks include 310 trials preceded by a practice section. The digits 1 to 9 appear in white on a black computer screen. In the SART block, digit 3 is the infrequent target, and the other digits (1, 2, 4–9) are frequent nontargets. Participants were required to respond by key press to digit 3 while ignoring all other digits. On the contrary, in the Control block, digit 3 is the frequent nontarget, and the other digits (1, 2, 4–9) are targets. Participants were required to respond by key press to all digits (1, 2, 4–9) except digit 3. In each trial, the digits are presented centrally on the computer screen in random order. Each digit is displayed for 250 milliseconds (ms), followed by a 900 ms duration mask composed of a cross (“+”) presented in the middle. The digits were presented in a predetermined and quasi-random manner, so the same digits did not cluster. A probe was randomly presented on the screen. The probe question was that “Was your mind wandering just now? If your mind was not wandering, please press 1; if you know your mind was wandering, please press 2; if you do not know your mind was wandering, please press 3.” The first question was to investigate whether participants’ attention was focused on the task. If the participants’ attention was focused on the task, then their mind was not wandering, and they would press 1. If their attention was not focused on the task, then their mind may be wandering, and the second and third questions would investigate their mind wandering. The two questions asked the participants how aware they were of where their attention was during the task. If they were aware, they would press 2; if they were unaware, they would press 3. Throughout the task, participants were seated in a comfortable chair, and were instructed to respond as rapidly and as accurately as possible. Experimental procedures and behavioral responses were collected using the E-prime software (Psychology Software Tools Inc., E-prime 2.0, Pittsburgh, PA, USA).

To measure the behavioral performance in SART, we calculated the following indicators: (1) Omission error, which refers to the error when the participants did not respond to the GO trial (target), and omission error ratio = omission error ÷ total number of target trials; (2) commission error, which refers to the error when the participants responded to the NOGO trial (nontarget) and commission error ratio = commission error ÷ total number of nontarget trials; (3) reaction time coefficients of variability (RTCVs), which is calculated by taking the standard deviation of the eight trials preceding each probe, divided by their mean; 4) response to probe questions, including ratio of “no mind wandering”, ratio of “know mind wandering”, and ratio of “not know mind wandering”.

### 2.5. The Math Task

The Math task was developed to investigate mathematical skills such as numerical and calculating capacity, which demanded cognitive abilities including sustained attention. In the current study, an experimental instruction and 16 lines of Arabic numbers were printed on a piece of A4 paper. There were 52 numbers in each line, ranging from 0–9. These numbers were randomly distributed with different weights, 0 had fewer occurrences than the other digits, for example. Two different arithmetic computation tasks were performed in the pre-test and post-test sessions. More concretely, in each line, it was totally different in both listed numbers and numerical orders. Nevertheless, for the whole task, the number of pairs of two adjacent integers adding up to 10 remained the same, which were 138.

Participants were seated in a comfortable chair, with adequate lighting as well as good writing conditions (i.e., a writing board underlying the paper). During the computation task, the 16 lines of numbers were printed on a piece of paper, with 52 numbers in each line. The participants were required to identify and mark all the pairs of two adjacent numbers whose sum equaled to 10. Throughout the task, participants were required to respond as rapidly and as accurately as possible line by line. In addition, the participants were not allowed to look back to the previous lines for a second check and revision of answers. Total time was recorded by a stopwatch.

To measure the behavioral performance in the Math task, we calculated the following indicators. (1) Overall accuracy (ACC); (2) overall response time (RT); and (3) calculation efficiency, which is overall RT divided by overall ACC (RT/ACC).

### 2.6. The Tactile Training

The tactile training is a custom-designed closed-loop adaptive fingertip manipulation task. The experimental devices consisted of a Six-Axis Force Sensor System (ATI Industrial Automation Inc., ATI Nano17, Apex, NC, USA), a pair of head-mounted earmuffs, an eyeshade, a Haptuator (Tactile Labs Inc., TL-002-14R, Montreal, QC, Canada), and a computer with a control software developed in C# language. Participants wore eye and ear masks to block out auditory and visual interference. All stimuli, including the feedback, were tactile stimuli. During the tactile training, the participants were instructed to press the force sensor button and keep the force at about 1.5 N using the index or middle fingers in both hands. If the force produced by the press is within the required range, the neck vibrator will vibrate to prompt the completion of the task. If the force is too large, the vibrator located on the corresponding finger will begin to vibrate to indicate the magnitude of the reduction of the force; if the force is too small, there is no feedback. The participants need to judge which finger may have a small force and make adjustments accordingly. The tactile training consisted of 4 blocks (80 trials in total), and the whole experiment in each day lasted approximately 40 min, including the set up and practice time. The real training time was about 32 min per day, and the total real training time was about 160 min.

### 2.7. EEG Acquisition and Preprocessing

To measure the brain activity during the SART, EEG was recorded with 128-channel HydroCel geodesic sensor nets and a GES 300 amplifier (Electrical Geodesic Inc., EGI, Eugene, OR, USA). Electrode Cz served as reference. Data was recorded at 250 Hz sampling rate using EGI’s NetStation 4.5.6 software. Impedances were kept below 50 kΩ, in accordance with the current guidelines proposed by the Society for Psychophysiological Research [25]. Data analysis for EEG channels was conducted using EEGLAB toolbox [26]. After administration of a 0.5 Hz high-pass finite impulse response filter and a 100 Hz FIR low-pass filter, the data was segmented according to the two conditions: target and nontarget conditions. For data segmentation, we used the beginning of the number stimuli as onset, and data were segmented into epochs with lengths from −1000 to 1000 ms with respect to the onset. We used the 50 Hz notch filter to remove the power interference. Independent components analysis was used to identify and remove components reflecting residual muscle activity, eye movements, blink-related activity, and other artifacts. Finally, the artifact-free epochs were grand averaged for all trials for all participants at Pz channel in each condition for ERP waveforms. Then the P300 and N200 amplitudes were computed by extracting the largest amplitude of averaged epochs in each participant in the 280 to 448 ms time periods (P300) and the 100 to 280 ms time periods (N200). We computed the P300 and N200 latencies for each participant by extracting the time point corresponding to the peak amplitude in the averaged epochs during the P300 and N200 time periods.

### 2.8. EEG Source Imaging

The preprocessed EEG data were then loaded into the Brainstorm toolbox [27] for the EEG source imaging analyses. Innovations in source imaging technology have transformed EEG from one-dimensional sensing or two-dimensional mapping to three-dimensional imaging for mapping of dynamically distributed brain activity, mainly from the cortex, with higher temporal (1 ms) and increased spatial (5–10 mm) resolution [28]. The availability of a dense array EEG provides an opportunity to sense the temporal and spatial distribution of electrical activity on the scalp. Numerous studies in psychiatry, neurosurgery, clinical neurology, and cognitive neuroscience have demonstrated the power of EEG source imaging in characterizing dynamic brain activity [28,29,30]. For EEG source imaging analysis, we first built a forward model using OpenMEEG BEM, and then computed the noise covariance matrices. Next, the standardized low-resolution brain electromagnetic tomography (sLORETA) algorithm was used for solving the inverse problem.

### 2.9. Statistical Analyses

Statistical analyses were performed using the SPSS software version 16.0 (IBM, Armonk, NY, USA). To examine the performance differences (e.g., omission error) within each group between pre-test and post-test sessions, we used the paired *t*-test. An analysis of covariance (ANCOVA) model was used to test for the between-group differences in behavioral and ERPs data following training (e.g., calculation efficiency during post-test session), while controlling for the pre-intervention levels, with the pre-test performance (e.g., calculation efficiency during pre-test session) as the covariates and group (trainees vs. non-trainees) as the independent variables. The ANCOVA model has been widely used in training and intervention studies [4,31]. Regarding the brain activation data, permutation *t*-test was used to examine the differences in the activation of different brain regions as measured by the Desikan-Killiany atlas [32]. The Desikan-Killiany atlas divides the whole brain surface into 68 brain regions. We used a false discovery rate (FDR) correction model [33] inserted in Brainstorm to adjust all pooled *p* values for controlling for multiple-comparisons of different brain regions when examining the differences in brain activation measured by EEG source imaging. All *t*-tests were 2-sided, with α = 0.05. We also calculated the Cohen’s *d* to estimate the effect size. If the value of Cohen’s *d* = 0.2 is considered a small effect size, 0.5 represents a medium effect size, and 0.8 represents a large effect size.

## 3. Results

### 3.1. Improved Behavioral Performance following the Tactile Training

**Figure 3:**
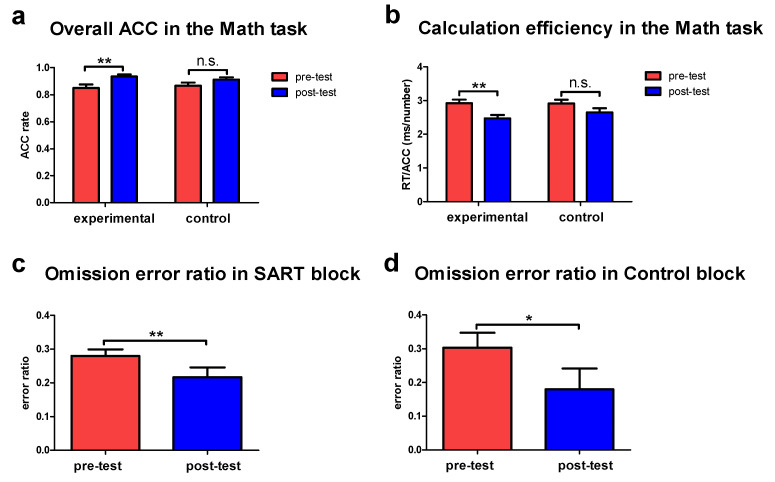
Improvements in sustained attention. (**a**,**b**) Shows improvements in overall accuracy (ACC) and calculation efficiency following training measured by the Math task. (**c**,**d**) Shows reductions in the omission error ratio following training in the SART block and Control block measured by SART. * *p* < 0.05, ** *p* < 0.01, and n.s. denotes not significant. Error bars are the standard error of the mean (s.e.m.).

Figure 3 shows the behavioral results as measured by the Math task and SART. We observed significant improvements in the behavioral performance following training. For the Math task, we found that trainees (participants in the experimental group) demonstrated significantly increased accuracy rate following training (*p* = 0.007, Cohen’s *d* = 0.96, Figure 3a), whereas the non-trainees did not show significant changes from pre-test to post-test sessions (*p* = 0.12, Cohen’s *d* = 0.56, Figure 3a). Furthermore, there were significant differences in accuracy rate between the trainees and non-trainees following training (*p* = 0.05, Cohen’s *d* = 0.46), as evaluated by the ANCOVA model. Moreover, we observed that trainees demonstrated lower RT/ACC scores (*p* = 0.004, Cohen’s *d* = 1.05, Figure 3b) following training, indicating better calculation efficiency. However, the non-trainees did not show the improved calculation efficiency from pre-test to post-test sessions (*p* = 0.11, Cohen’s *d* = 0.56, Figure 3b). Regarding the between-group differences in the calculation efficiency, the ANCOVA model revealed significant differences between the trainees and non-trainees following training when controlling for the pre-test calculation efficiency (*p* = 0.04, Cohen’s *d* = 0.99). For the SART, trainees demonstrated significantly decreased omission error ratio in the SART block following training (*p* = 0.01, Cohen’s *d* = 0.65, Figure 3c). Furthermore, we observed significantly decreased omission error ratio in the Control block following training (*p* = 0.03, Cohen’s *d* = 0.59, Figure 3d).

However, there were no significant differences in the commission error ratio in the SART block (*p* = 0.15, Cohen’s *d* = 0.36) and Control block (*p* = 0.85, Cohen’s *d* = 0.11) following training. RTCVs also did not show significant changes in the SART block (*p* = 0.76, Cohen’s *d* = 0.07) following training. For the real-time probe questions about mind wandering inserted in the SART, trainees reported significantly less mind wandering (more “no mind wandering”) following training (*p* = 0.03, Cohen’s *d* = 0.61). There is also more “know mind wandering” (*p* = 0.04, Cohen’s *d* = 0.45) following training, indicating the trainees’ awareness of their current state. Moreover, there was no significant difference in “not know mind wandering” (*p* = 0.12, Cohen’s *d* = 0.62) following training.

### 3.2. Increased P300 Event-Related Potentials (ERP) Amplitudes following the Tactile Training

**Figure 4:**
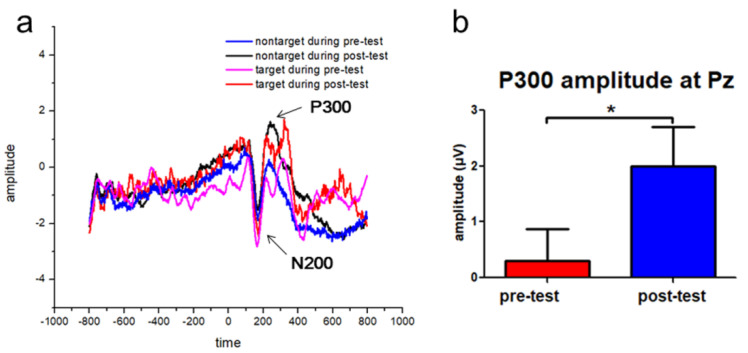
Changes in the event-related potentials (ERPs) at Pz channel following training. (**a**) N200 and P300 components were shown in both target and nontarget conditions. Time in milliseconds, and amplitude in μV. (**b**) Shows changes in P300 amplitude for the target stimuli following training. P300 amplitude was significantly increased following the tactile training (*p* = 0.03), whereas there was an equivocal effect for the N200 amplitude between the pre-test and post-test sessions (*p* = 0.07). The time period of P300 was 280–448 ms, and the time period of N200 was 100–280 ms. * *p* < 0.05, ** *p* < 0.01, and n.s. denotes not significant. Error bars are the standard error of the mean (s.e.m.).

The ERP analysis showed obvious N200 and P300 ERP components in the target and nontarget conditions in the Pz channel (Figure 4a), which was consistent with previous studies [34,35,36]. Paired *t*-tests showed that trainees demonstrated significantly increased P300 amplitude following the tactile training (*p* = 0.03, Cohen’s *d* = 0.72, Figure 4b). Moreover, there was an equivocal effect for the N200 amplitude between pre-test and post-test sessions (*p* = 0.07, Cohen’s *d* = 0.57). Regarding the latencies, there were no significant changes in the latencies of N200 and P300 components following training.

### 3.3. Increased Brain Activation following the Tactile Training

**Figure 5:**
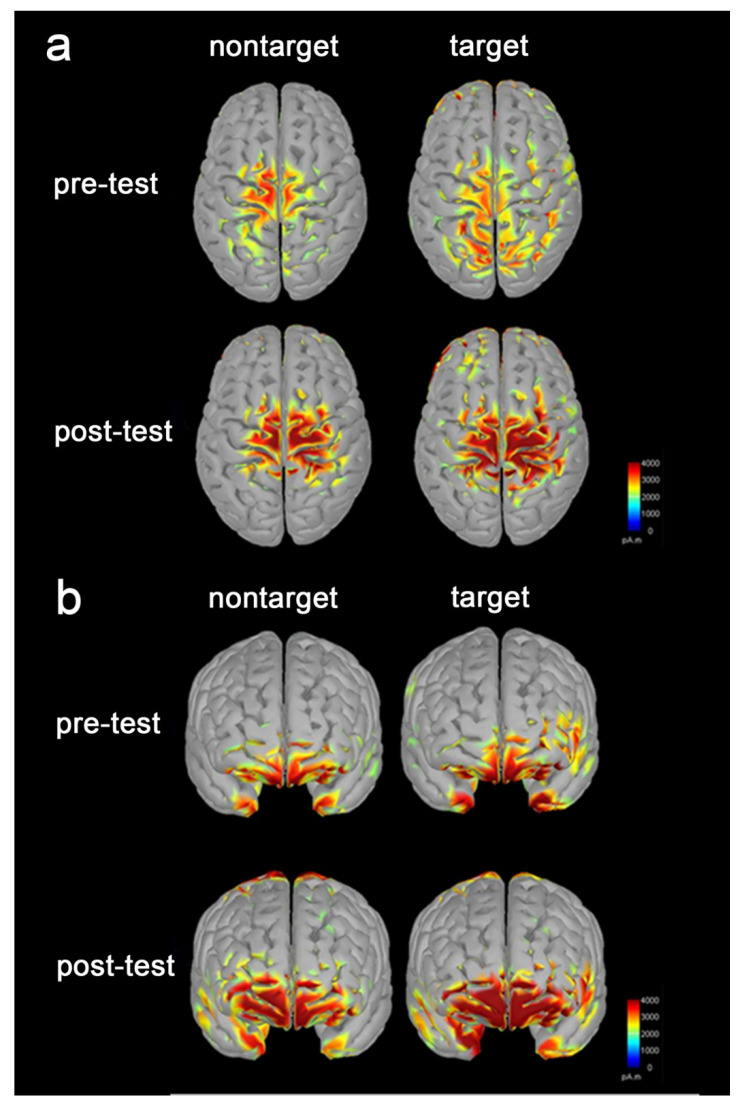
Changes in brain activation during P300 time periods following training. (**a**,**b**) Shows that the tactile training significantly increased the brain activation in the prefrontal and sensorimotor regions during the P300 time periods (280–448 ms). Degree of activation in pA.m, ranging from 0 to 4000.

Figure 5 demonstrates the EEG source imaging results of nontarget and target conditions during both pre-test and post-test sessions. Because of the high temporal resolution of EEG, we were able to compute the brain activation during P300 time periods (280 milliseconds to 448 milliseconds). Activation regions were mainly located in the prefrontal and sensorimotor cortex in both hemispheres. The trainees showed significantly increased brain activation in the frontal pole, lateral orbitofrontal cortex, medial orbitofrontal cortex, superior parietal cortex, primary motor cortex, premotor and supplementary cortex, and primary somatosensory cortices following training (*p* = 7.75 × 10^−8^, FDR correction). The data was given the significant difference in brain activation in all those regions between pre-test and post-test sessions.

## 4. Discussion

The aim of this study was to evaluate the effect of a short-term tactile training on the visual sustained attention in young adults. There were three main findings. First, we observed improvements in behavioral performance following the tactile training, including the increased ACC and calculation efficiency measured by the Math task, decreased omission error ratio in the SART block and Control block, and less mind wandering measured by the SART real-time probes. Second, N200 and P300 at the Pz channel were identified in the target and nontarget stimuli measured by SART, and we also found significantly increased P300 amplitude following training. Third, we found stronger brain activation in the prefrontal and sensorimotor areas in the P300 component following training. These findings are discussed below. In the study, the tactile training may induce the observed learning and neural plasticity effects, which lasted one day after five days of 40-minute daily training sessions.

The first main finding is that the closed-loop tactile training contributed to behavioral improvements in visual sustained attention. Consistent with previous studies [17], we found significantly increased ACC and calculation efficiency following training measured by the Math task, which suggests that trainees may be more effective in identification of digit pairs using a more central scan with sustained attention. Furthermore, we observed that the omission error ratio was significantly decreased in the SART block and Control block following training, indicating less attention distraction and superior sustained attention. Omission error measured by the SART is related to the failure to respond to the target (GO) stimulus, and seems to reflect the capability to sustained attention across trials [37]. Previous studies have interpreted the omission error ratio as a separation from task participation and thus reflects attention distraction or attention lapse [38,39]. Omission error ratio has been shown to be increased in children with attention-deficit/hyperactivity disorder (ADHD) [39,40], and patients with traumatic brain injury [41]. In the present study, we found that participants demonstrated significantly decreased omission error ratio following the tactile training. Therefore, our results suggest that the tactile training can decrease the omission error, thus contribute to enhanced sustained attention. Moreover, using the real-time probes inserted in the SART, we found that participants illustrated less mind wandering following the tactile training. Mind wandering, defined as a shift of attention away from a primary task toward internal information [42], occupies approximately 46.9% of our waking life [43,44]. Previous neuroimaging studies have found that mind wandering is associated with the recruitment of default network and executive network [43]. Though mind wandering is an extraordinary evolutionary achievement that enables people to plan, learn and reason, it has an emotional cost. A study of 2250 adults regarding the real-time reports of mind wandering found that people were less happy when they were mind wandering than they were not, suggesting that a wandering mind may be an unhappy mind [44]. Therefore, our findings of less mind wandering in the trainees indicate that the short-term tactile training can reduce mind wandering in young adults and help them concentrate more on the current task.

The second main finding is that trainees demonstrated increased P300 amplitude at Pz channel following the five-day tactile training as measured by the SART. Consistent with previous studies [34,35,36], we identified N200 and P300 ERP components at Pz in the SART. Furthermore, statistical analysis revealed significant improvements in the P300 amplitude and an equivocal difference in the N200 amplitude following training. N200 and P300 are usually associated with the processes of attention [45]. According to a response model, the amplitudes of N200 and P300 indicate that cognitive control processes reflected by these neurophysiological associations of the response inhibition sub-processes are reinforced with a response pattern characterized by automatic responses and frequent impulse errors [46,47]. Reduced P300 amplitude is an indicator of potential broad neurobiological vulnerability [48]. The reduced P300 amplitude has been reported in Huntington’s disease [34], attention deficit hyperactivity disorder (ADHD) [49], schizophrenia [30], and major depressive disorder [50]. Here, we found increased parietal P300 amplitude following training. Two possible reasons may account for this phenomenon. One explanation is that the trainees may be more automatic to process the visual stimuli following training. The neural changes of P300 amplitude have been considered to reflect the task difficulty [45]. The P300 amplitude decreases when the task is more difficult [51,52]. When the task is easy, participants become more automatic in information processing and have larger parietal P300 amplitude [36,45]. Previous studies have interpreted the increased P300 amplitude in go/no-go tasks as reflecting more rapid and automatic response-inhibition processes [36,53,54,55]. Another explanation for the increased P300 amplitude is that trainees may allocate more attentional resources for the visual stimuli following training. Parietal P300 amplitude has been associated with attentional resource allocation [56]. Previous studies have found that for tasks requiring greater amount of attention resources, the P300 amplitude is decreased [56,57]. Increased P300 amplitude at Pz is like to reflect more effective attentional resources allocation available for the SART task [45,56]. Therefore, the findings of increased P300 amplitude at Pz channel may either reflect more automated information processing or more effective attentional resources allocation following training.

The third main finding is that stronger brain activation was shown in the prefrontal and sensorimotor areas following training. This is in line with previous SART studies [58]. Neuroimaging studies have related the brain activation in the frontal regions (e.g., premotor cortex) in SART to sustained attention and processing of task related information [59,60]. In the present study, we observed larger extent of brain activation in the prefrontal and sensorimotor areas following training. The training-induced neural changes may be due to the faster, more automatic information processing in task-specific neural circuits [61]. Training is usually associated with decreased brain activation in the prefrontal cortex [62,63], a key brain area underlying cognitive control [61,64]. However, we found increased activation in the prefrontal cortex following training, which is inconsistent with the automatic processing hypothesis. Therefore, the enhanced behavioral performance is not likely attributable to more automated information processing following the tactile training. Another possible reason for the increased activation is that participants may be more effective in the attentional resources allocation for information processing following training. Decreased brain activation in the prefrontal areas has been demonstrated in no/no-go tasks in children with ADHD [65], indicating poorer attentional resources allocation [66]. Human information processing capacity is limited by attention resources [67]. When performing the time-critical tasks (e.g., the SART), participants recruit shared attentional resources across the sensory modalities residing in the frontal lobe [67,68]. Stronger activation in the prefrontal and sensorimotor areas has been associated with more effective attentional resources allocation [69,70]. Consistent with previous sensorimotor training studies [71], we observed stronger brain activation in prefrontal and sensorimotor areas following training. Therefore, our results suggest that the young adults may be more effective for attentional resources allocation following training.

Consistent with previous studies [17,72], we showed that the sustained attention may be modality independent, since the tactile training improved the visual sustained attention. Previous behavioral studies found that the force control training with pure haptic feedback could enhance visual focused attention [17], indicating the modality independence of attention. Furthermore, neuroimaging studies found that attention could spread across modalities and that visual attention could modulate neuron activity in auditory cortex [72]. In the present study, we observed significant improvements in visual sustained attention following the tactile training, supporting a cross-modal transfer training effect. Our study was primarily focused on the practical effectiveness of the training, but it also appeared to indicate a more “basic science” point that sustained attention may be “modality independent”.

Several limitations in the study are worth consideration. While the number of participants was adequate to detect statistically significant differences in the behavioral and neurophysiological changes induced by the tactile training, larger sample size might increase the statistical power. Another limitation is that the participants in the control group did not perform the SART. Previous studies have found that there were no significant differences in the SART performance for participants who received no training or placebo interventions [73]. Furthermore, in the present study, the experimental group received five days of tactile training, while the control group received no training. A habituation effect to the context (neural or sensory adaptation) may happen during the training session. If we have included a placebo group in which participants perform a non-tactile task, our conclusions of enhanced sustained attention following the tactile training would be better supported. Further studies taking in account these factors would make the conclusions much stronger in relation to the role of the tactile training to the improvement of sustained attention. A further study investigating the persistent effect of training (e.g., testing again three months/one year later) would help to better pinpoint the neural basis of various behaviors and the training-related plasticity. Moreover, identifying and verifying the biomarkers in an independent sample set might better test the generalization ability of these biomarkers.

## 5. Conclusion

In conclusion, behavioral assessments and neurophysiological measures were used to investigate the tactile training effect on sustained attention in young adults. We found that the tactile training can enhance visual sustained attention. Trainees demonstrated increased ACC and calculation efficiency, decreased omission error ratio, increased P300 amplitudes, and stronger activation in the prefrontal and sensorimotor areas following training. These results indicate that trainees may allocate their attentional resources more effectively following training. The findings may help illustrate the potential use of tactile training in sustained attention enhancement across sensory channels and cognitive impairment amelioration.

## Figures and Tables

**Table 1:** Demographics between the experimental and control groups.

	Experimental Group (*n* = 17)		Control Group (*n* = 17)			
	Mean	SD	Mean	SD	*p* Value	Cohen’s *d*
Gender (M/F)	8/9	—	8/9	—	—	—
Age (years)	22.5	1. 6	22.9	1.1	0.9	0.3
Education (years)	16.5	0.9	16.7	1.2	0.5	0.2
Daydreaming frequency subscale (scores)	33.9	7.2	33.7	5.8	0.1	0.04

Note: M and F denote male and female. The length of education was calculated starting the first grade of primary school.
